# MRI-based habitat imaging in cancer treatment: current technology, applications, and challenges

**DOI:** 10.1186/s40644-024-00758-9

**Published:** 2024-08-15

**Authors:** Shaolei Li, Yongming Dai, Jiayi Chen, Fuhua Yan, Yingli Yang

**Affiliations:** 1 Institute for Medical Imaging Technology, Ruijin Hospital, Shanghai, 201800 China; 2 School of Biomedical Engineering, ShanghaiTech University, Shanghai, 201210 China; 3 Department of Radiation Oncology, Ruijin Hospital, Shanghai, 201800 China; 4 Department of Radiology, Ruijin Hospital, Shanghai, 201800 China

**Keywords:** Habitat imaging, Tumor heterogeneity, Cancer treatment

## Abstract

Extensive efforts have been dedicated to exploring the impact of tumor heterogeneity on cancer treatment at both histological and genetic levels. To accurately measure intra-tumoral heterogeneity, a non-invasive imaging technique, known as habitat imaging, was developed. The technique quantifies intra-tumoral heterogeneity by dividing complex tumors into distinct sub- regions, called habitats. This article reviews the following aspects of habitat imaging in cancer treatment, with a focus on radiotherapy: (1) Habitat imaging biomarkers for assessing tumor physiology; (2) Methods for habitat generation; (3) Efforts to combine radiomics, another imaging quantification method, with habitat imaging; (4) Technical challenges and potential solutions related to habitat imaging; (5) Pathological validation of habitat imaging and how it can be utilized to evaluate cancer treatment by predicting treatment response including survival rate, recurrence, and pathological response as well as ongoing open clinical trials.

## Background

Intra-tumoral heterogeneity is one of the characteristics of malignant tumors, which can cause differences in tumor growth rate, invasion and metastasis, drug sensitivity, and prognosis. The discovery and development of tumor driver genes and targeted drugs have opened the door to the hope of overcoming tumors, but the existence of heterogeneity has made tumor treatment fall into a dilemma that is difficult to overcome [1]. Intra-tumoral heterogeneity has been widely thought to drive tumor adaptation and resistance over the course of treatment in cancer patients, compromising the outcomes of treatments [2]. Usually, this phenomenon leads to severe consequences such as rapid tumor progression, failure of treatment, and eventually lowers the survival rate of patients [3]. In order to effectively address Intra-tumoral heterogeneity, especially in the context of tumor recurrence, progression, and evolution, it is essential to have a comprehensive understanding of its causes and phenotypic variations for personalized treatment. Personalized treatment plans are now a crucial aspect of precision medicine, and to develop such plans, it is necessary to have non-invasive tools for measuring intra-tumoral heterogeneity. At a microscopic level, heterogeneity can be explained by different groups of cells known as tumor niches, which share similar characteristics within distinct micro-environments. This heterogeneity is characterized by gene expression patterns, which also have a significant impact on treatment response [4]. However, measuring spatial heterogeneity at a macroscopic level has been challenging, and there has been a lack of reliable methods to achieve this for a long time.

In the past few decades, clinical radiotherapy has been undergoing a considerable advancement, especially the technical developments in dose delivery which is able to deliver certain doses with extreme precision, enabling broadly personalized radiation therapy. Conventionally, radiation treatment regimens are formulated based on the average results of large clinical trials, with limited individualization, and no adjustments in dose or dose fractionation are made based on the individual clinical response of individual patients. Modern radiotherapy, on the other hand, has the potential to start a new radiotherapy paradigm to individualize each patient’s treatment. The development of advanced radiotherapy techniques such as intensity-modulated radiotherapy, image-guided radiotherapy, and online adaptive radiotherapy have revolutionized clinical radiotherapy and enabled personalized treatment to maximize patient response [5]. Advances in radiotherapy technology have further highlighted the importance of an intra-tumoral heterogeneity measurement technique.

With the advancements in medical imaging techniques, there are now effective tools available for the detection of spatial heterogeneity. Magnetic resonance imaging (MRI) is extensively used for guiding treatment and can also assist in non-invasively measuring the characteristics of the tumor micro-environment (TME). Various groups of researchers have explored the feasibility of MRI-based measurements of the micro-environment. Some groups have employed radiomics-based approaches to analyze quantitative features extracted using high-throughput computer techniques that describe both healthy and tumor structures [6, 7]. Despite the extensive research conducted in radiomics, only a few studies have successfully translated these findings into clinically useful tools [8–10]. Habitat imaging based on multi-parametric MRI is currently gaining attention for its ability to measure tumor heterogeneity in an explainable way. By partitioning the micro-environment into distinct sub-regions that exhibit similar characteristics, habitat imaging allows for the visualization of the tumor micro-environment and the monitoring of longitudinal changes. This method has the potential to provide insights into the distribution and evolution of intra-tumoral heterogeneity.

In this review, we examine the current utilization of habitat imaging in the field of oncological therapy, highlighting the technical details of habitat imaging as a non-invasive technique for assessing the heterogeneity within tumors. A number of clinical and technical studies about habitat imaging are collected. We primarily focus on studies that used MRI-based imaging biomarkers although some studies with positron emission tomography (PET)- and computed tomography (CT)- based imaging biomarkers are also mentioned to explain habitats generation methods and how to combine habitat imaging with radiomics in the next sections. Section 2 describes the methodology of this review. In Sect. 3, we summarize frequently employed biomarkers in habitat imaging, aiding in precise partition in a physiological manner. In Sect. 4, we review the habitat imaging techniques based on different clustering methods. There are also efforts trying to combine radiomics with habitat imaging in various ways as shown in Sect. 5. In Sect. 6, we discuss the existing technical obstacles. Furthermore, studies focusing on understanding the link between imaging and pathological or genetic information to validate imaging habitats and an overview of current applications of habitat imaging in cancer treatment especially radiotherapy were included in Sect. 7.

## Methodology

The articles from 2015 to 2023 were collected by searching on the PubMed database using the keywords “habitat imaging”, “MRI”, and “cancer treatment” to get 72 results. The results were reviewed manually to remove the irrelevant articles following these exclusion criteria: (1) review articles; (2) case reports and case series; (3) irrelevant study design. A total of 30 articles were selected, including 3 preclinical studies and 27 clinical studies. We have comprehensively reviewed the objectives, methodologies, and disease sites addressed in these articles, and provided an analysis of their respective strengths and weaknesses. While brain cancer serves as a prominent subject of study, the methodology of habitat imaging is versatile and can be extended to a spectrum of medical conditions. Some ongoing trials from clinicaltrials.gov are also included.

## Imaging biomarkers

Imaging biomarkers play a crucial role in habitat imaging, providing valuable information for TME. A biomarker can be defined as “an indicator of normal biological processes, pathogenic processes, or biological responses to an exposure or intervention, including therapeutic interventions” [11]. These biomarkers are instrumental in various diagnostic and treatment evaluation procedures. Specifically, imaging biomarkers can be derived from medical imaging modalities such as MRI, PET, and CT. Among all medical imaging modalities, MRI is the most versatile and can be used to explore structural, physiological, and functional information by manipulating the MRI pulse sequences. Historically, structural MRI biomarkers, including the signal intensities from T1- and T2-weighted imaging, have been widely used. Nevertheless, the scientific community has increasingly turned its attention to quantitative MRI (qMRI) biomarkers, recognizing their superior capability to delineate the metabolic dynamics within tumors non-invasively. Among the qMRI sequences routinely employed, diffusion-weighted MRI (DWI) stands out for its ability to measure the diffusivity of water molecules, while dynamic contrast-enhanced MRI (DCE-MRI) excels at documenting critical parameters such as vascular permeability. Table 1 contains an overview of widely utilized qMRI-derived biomarkers, specifically highlighting those that are indicative of diffusion and perfusion properties of various tissues. Each of them provides unique information about tumor biological characteristics in a distinctive manner. Habitat imaging is able to use imaging biomarkers to delineate distinct sub-regions by clustering pixels with the same biological and physical characteristics within a tumor [12]. Traditionally, clustering method have been applied to isolate individual biomarkers for straightforward tasks. However, the complexity of current advanced tasks is better addressed by integrating multiple biomarkers, taking into account their diverse attributes across various dimensions. For example, the positive predictive value (PPV) for glioblastoma recurrence has been notably enhanced by 10.3% when overlapping biomarkers [13].

**Table 1:** Examples of MRI-based biomarkers used for habitat imaging

Biomarkers	MRI Sequence	Descriptions
Apparent diffusion coefficient (ADC)	DWI	The measurement of apparent water diffusion of DWI. Following the formula *S*(*b*) = *S*0*e* − *bADC*, this value measures the relative decreases of transverse magnetization because of the dephasing caused by the additional diffusive gradients. Quantitatively, the measurement is given by the slope of the line plotting MRI signals and b-value. (units *mm*^2^ /*s*).
Volume transfer constant (*Ktrans*)	DCE	The most important parameter of DCE-MRI. *Ktrans is* a parameter that reflects the efflux rate of contrast agents into the extravascular-extracellular space and is commonly used to measure vascular permeability. (unit *min* ^− 1^)
Extravascular extracellular space fractional volume (*ve*)	DCE	This parameter is defined as the extravascular extracellular space (EES) per unit volume of tissue. *ve* is dimensionless, ranging from 0 to 1. It reflects how much amount of “room” is there in the tissue to accumulate contrast agents.
Fractional plasma volume (*vp* )	DCE	Like *ve*, *vp* is the fraction of plasma volume and therefore is also dimension- less. It is usually small in many lesions but in some high vascular tumors, it can reach a value that cannot be ignored.
Rate constant (*kep* )	DCE	*kep* determines the rate for contrast agent from EES back to the vascular system. It is defined by the ratio of *kep* = *Ktrans*/*ve*. (unit *min* ^− 1^)
Cerebral blood volume (CBV)	ASL DCE	CBV represents the amount of blood per unit brain tissue. It is usually calculated from the area under the concentration-time curve, which is derived from the intensity time curve generated by perfusion MRI. (unit *ml*/100*g*)

By employing multi-biomarkers, habitat imaging overcomes the limitations inherited to individual imaging biomarkers in terms of sensitivity. This strategy enables the comprehensive assessment and monitoring of a range of physiological processes, providing an accurate and non-invasive depiction of the tumor’s molecular profile. Therefore, choosing the correct biomarkers is an important step for habitat imaging. As an example, researchers from University of Washington [14] used PET to identify the hypoxia region in glioblastoma that is usually resistant to radiotherapy and chemotherapy and they found that higher volume size and intensity of hypoxia is highly associated with shorter time to progression (TTP) and poorer survival rate. Another group from Asan Medical Center [15] used a combination of T1- and T2-weighted MRI intensity, apparent diffusion coefficient (ADC), and cerebral blood volume (CBV) to enhance the sensitivity of a single biomarker. This approach was employed for assessing and monitoring multiple physiological aspects and localizing viable tumor tissue in patients with brain metastases post stereotactic radiosurgery (SRS).

### Radiomics features as biomarkers

Beyond the conventional biomarkers, the implementation of radiomics methodology facilitates the extraction of quantifiable data, enriching the diagnostic process with a deeper layer of measurable insights. Radiomics has been extensively utilized to generate an extensive range of characteristics from the tumor, creating a unique dataset that significantly enhances diagnostic accuracy, prognostic assessment, and treatment outcomes estimation [16]. In Sect. 5.1, radiomics analyses on tumor sub-regions as an integration into habitat imaging will be discussed. Although this method is primarily aimed at uncovering the link between radiomics features generated using only voxels within tumor sub-regions and the overall outcomes of cancer therapy, there is still unexplored potential within these sub-regions regarding the underlying TME heterogeneity [17]. Further research could incorporate voxel-wise radiomics feature maps as the input biomarkers for generating habitats, utilizing a combination of imaging techniques to enhance the delineation of tumor sub-regions. In Sect. 5.2, radiomics features used as imaging biomarkers for generating habitats will be discussed in more details.

## Generation of imaging habitats

Habitat imaging visualizes tumor heterogeneity by identifying different habitats. Imaging habitats are distinct spatial regions with shared imaging characteristics due to their unique intrinsic cell populations and/or TME conditions. Typically, the construction of imaging habitats is facilitated by clustering techniques that aggregate similar data points, frequently derived from a diverse array of imaging biomarkers. Many researchers apply automated clustering methods based on machine learning algorithms or digital image processing tools. Some other analyses are still based on manually selected habitats. In this section, we provide an overview of common clustering methods for habitat generation and some techniques for optimizing habitat generation.

### Automatic habitat clustering methods

**Fig. 1:**
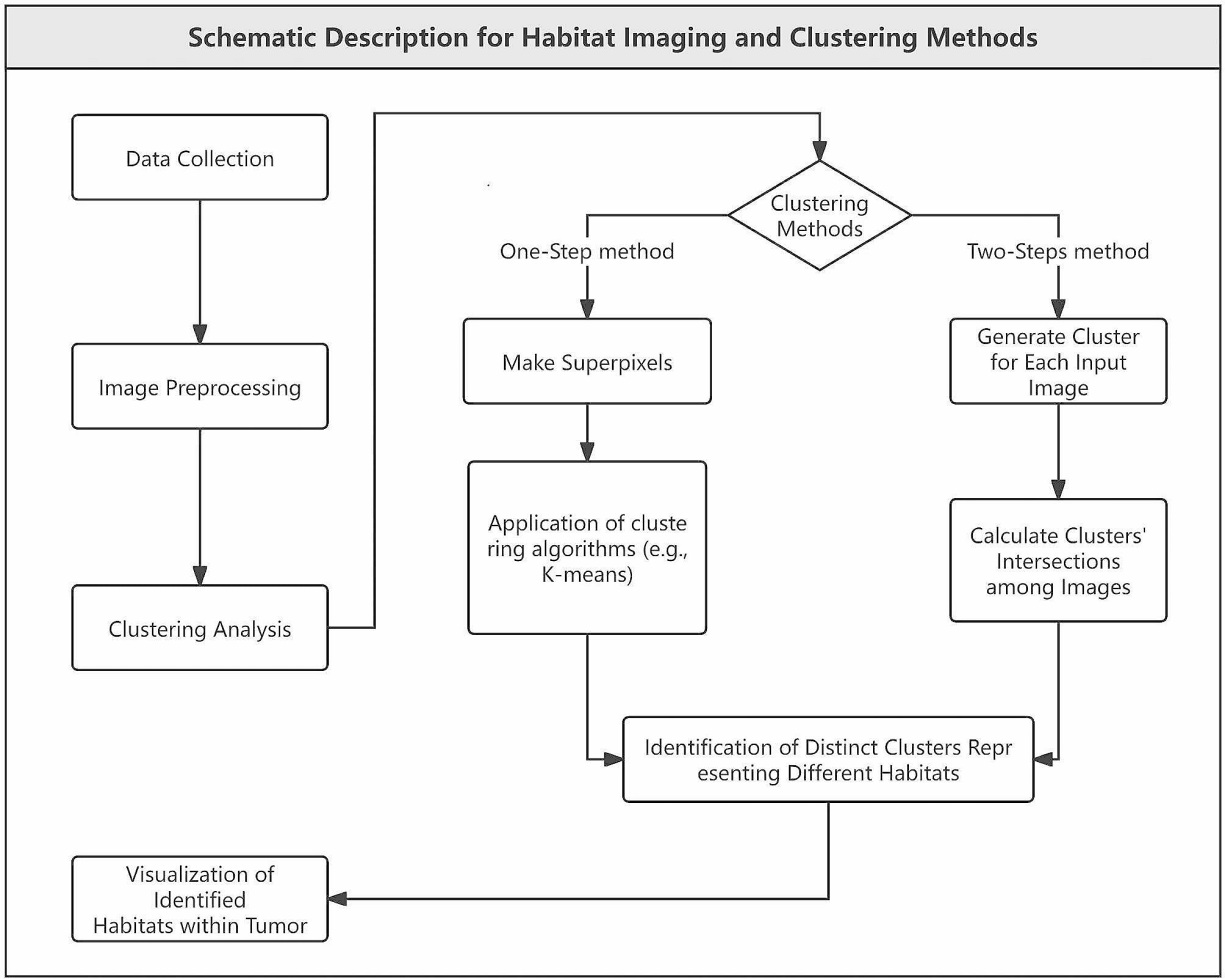
A schematics of habitat imaging analysis

**Fig. 2:**
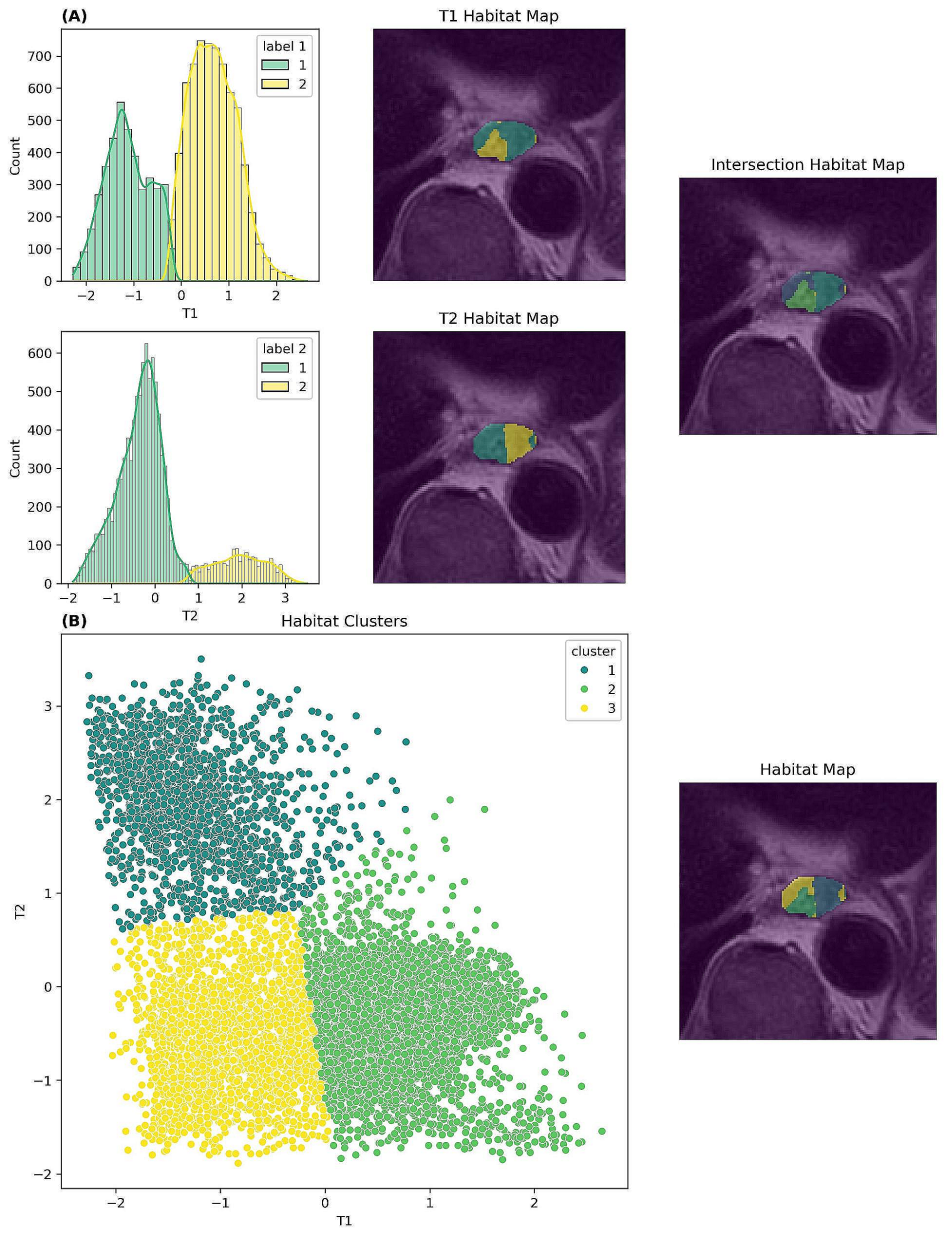
An example of habitat imaging based on T1- and T2-weighted images of an esophageal cancer patient using two-steps method (A) and one-step method (B). The region of interest is the gross tumor volume. Both T1 and T2 images are preprocessed using central normalization. (A) Each of T1 and T2 images generates 2 clusters (1 and 2) derived from histogram and 4 habitats (11, 12, 21, and 22) created by intersections between T1 habitat map and T2 habitat map for the final habitat map; (B) The scatter plot (left) and map (right) share the same color map to identify the 3 clusters using K-mean algorithm

A variety of clustering methods are employed in the studies of habitat imaging, encompassing primarily two distinct approaches. One approach involves the utilization of a machine learning-based clustering algorithm as a “one-step” approach, commonly exemplified by the K-means clustering technique. This technique amalgamates and organizes multi-dimensional biomarkers to identify various groups through overlapping and clustering processes [18, 19]. The other approach, conversely, is more traditional in nature and involves two steps. In this alternative method, voxels are partitioned based on each single biomarker, and combinations of these clusters from multiple biomarkers are subsequently employed to discern different habitats [20]. The predominant approach involves calculating the intersection of all habitat maps. For each voxel, it is assigned to a specific cluster on every biomarker map. The ultimate cluster to which it belongs is determined by the collective set of clusters across all maps. As shown in Fig. 2A, T1 and T2 images both generates 2 clusters, 1 and 2. Therefore, we get 4 combinations of intersection (11, 12, 21, and 22) as the final result. The process to generate habitats of both approaches are shown in Fig. 1 and samples are shown in Fig. 2.

The “one-step” approach has gained popularity in recent years due to the increased accessibility and usability of machine learning tools. A study conducted by researchers from Asan Medical Center focused on the application of habitat imaging to the progression-free survival (PFS) estimation of glioblastoma patients after concurrent chemoradiotherapy (CCRT) [21]. Employing K-means clustering as an automated approach to imaging habitat generation, the researchers were able to identify three distinct habitats: hypervascular cellular, hypovascular cellular, and nonviable tissue, using CBV and ADC data. They observed that an increase in volume size of both hypervascular and hypovascular cellular habitat was indicative of tumor progression, suggesting its potential as a useful predictor of clinical outcomes. The effectiveness of the “one-step” approach was further supported by a study conducted by a group from Ajou University School of Medicine [22]. This study focused on predicting tumor recurrence of brain metastases. Beyond the physiological features of CBV and ADC, the researchers expanded their analysis to include structural features such as contrast-enhanced T1- and T2-weighted MRIs, thereby delineating a comprehensive set of six distinct habitats. Researchers found that an increase in volume size of the hypovascular cellular habitat, characterized by low ADC and low CBV, was associated with a higher risk of recurrence. Furthermore, this habitat provided crucial insights about the recurrence location, which could guide future treatment strategies for patients.

The traditional “two-steps” method is still widely used in many studies [23, 24]. An initial step in clustering involves the utilization of simple techniques, such as partitioning voxels based on their intensity values. Stringfield O. et al. [25] and Weinfurtner R.J. et al. [23] conducted voxels clustering to analyze quantitative changes in intra-tumoral habitats, specifically focusing on the differentiation between low and high-intensity classes using Otsu thresholding [26]. Lee J. et al. [24] from the University of Texas employed Gaussian mixture for clustering. The habitats were then generated by calculate intersections among the habitats from single biomarkers. Both groups have identified that the volume fractions of habitats were associated with treatment response, thus demonstrating significant predictive values.

Some Researchers have developed more advanced clustering techniques. Wu J. et al. [27] have successfully developed an imaging signature for predicting progression-free survival (PFS) in patients with oropharyngeal squamous cell carcinoma (OPSCC). This achievement leverages a robust consensus clustering approach, which is designed to delineate distinct sub-regions, or ‘habitats,’ within the tumor based on an analysis of PET and CT imaging data. Their method was a 2-steps clustering process: individual-level clustering based on parametric maps followed by a population-level consensus clustering. Consensus clustering, alternatively known as cluster ensembles or aggregation of clustering, is an advanced methodology that amalgamates various clustering algorithms. This synthesis aims to yield a unified consensus clustering that serves as a more accurate representation than the individual clustering methods typically employed. Beyond the straightforward application of a solitary clustering method, consensus clustering was used to delve into similarities across or within patient groups, thereby revealing consistent patterns that emerge from an ensemble of clustering methodologies.

### Manually selected methods

Studies have also been conducted to generate habitats through manual selection of particular sub-regions [28, 29]. Beig N. et al. [28] examined 936 3D-radiomic characteristics that were derived from various sub-regions of the tumor (necrotic-core, enhancing tumor, peri-tumoral edema) based on multi-parametric MRIs. The necrotic-core was the low intensity on Gd-T1 images; enhancing tumor was the high signal intensity area compared to the pre-contrast T1 image; peri-tumoral edema was identified by comparing T2 and T2-FLAIR. The thresholds for segmentation were manually annotated by exports. Verma R. et al. [29] also formulated an radiomics risk score (RRS) based on the sub-regions of necrotic core, enhancing tumor, and FLAIR-hyperintense subcompartments. The sub-regions of the tumor which these studies’ analyses were based on were manually selected by researchers. However, it is important to note that the primary focus of these studies was not on how they selected these sub-regions.

### Optimization of habitat generation

Researchers are committed to advancing cluster generation algorithms to create more precise and insightful clustering outcomes. Neto da Silva et al. [30] developed two approaches, namely the molecular texture descriptor (MTD) and pathophysiological texture mapping (MPT), to improve habitat generation. MTD boosts the contrasting agent’s area while MPT generates habitats based on the updated generation from MTD. This novel technique not only demonstrates the ability to identify breast tumors with 100% accuracy but also enables the classification of tumor malignancy. Another research team, led by Xing et al. [31], introduced a novel approach to classifying habitats by employing a probability distribution model associated with reference tissue. In comparison to image-based methods for habitat generation, this technique demonstrates qualitative consistency with pathological observations. Additionally, Tar et al. [32] utilized habitat imaging to detect tumor response to drug and radiation therapy in an innovative way. With the aid of the linear Poisson modeling, their approach significantly reduced the number of animals needed for multiple therapeutic interventions, thereby enhancing information acquisition from preclinical imaging.

### Summary

In brief, recent trials on habitat imaging in treatment evaluation tasks have yielded fruitful outcomes in various domains including risk mitigation, survival and recurrence estimation by employing different clustering methodologies. However, there is a dearth of studies that compare the efficiency and accuracy of different clustering methodologies. It is crucial for researchers to strategize while choosing the most optimal methodologies for their investigations. Nevertheless, some studies have explored new habitat generation methods such as consensus clustering. Additionally, researchers are examining techniques for optimizing data clustering. These investigations offer potential research avenues for future explorations. Table 2 presents an overview of all studies mentioned in Sect. 4.

**Table 2:** List of studies of the habitat generation methods mentioned in Sect. 4. simDWI = a quantitative surrogate for high-b-value DW-MRI.

Author(s)	Year	Intensity Normalization	Biomarkers	Clustering Method	Habitat Generation Method	Data Availability	Analysis Tool Availability
Slavkova, K.P [18]	2023	Yes	ADC, Ktrans, ve, kep	K-means, Agglomerative	1-step approach to generate 3 habitats	Self-prepared from scanning mice; request with reasons	Matlab code not publicly available
Lee, DH [22]	2023	Yes	CET1, T2, ADC, CBV	K-means	1-step approach to generate 3 habitats for each condition	Database from Asan Medical Center; request with reasons	Brain extraction code: https://github.com/MIC-DKFZ/HD-BET;Lesion segmentation: https://github.com/MIC-DKFZ/nnUNet; k-mean clustering by scikit-learn
Kazerouni, A.S [19]	2022	Yes	ADC, Ktrans, ve, kep	Agglomerative	2-steps approach to generate 4 habitats	Self-prepared from scanning mice; request with reasons	R code not publicly available
Tar, P. et al. [32]	2022	No	ADC	Linear, Poisson Modelling	N/A	Self-prepared from scanning mice; request with reasons	LIFEx software (https://www.lifexsoft.org/) and ITK-SNAP (http://www.itksnap.org/pmwiki/pmwiki.php); Python code not publicly available
Weinfurtner RJ [23]	2022	No	DCE, T1	Otsu	2-steps approach to generate 8 habitats based on degree of contrast enhancement	Recruited; trial NCT03137693	Segmentation by Healthmyne software; analysis by Social Science Statistics software
Park, JE [21]	2021	Yes	CET1, FLAIR, ADC, nCBV	K-means	1-step approach to generate 3 habitats	Recruited; ClinicalTrials.gov NCT02619890	R code not publicly available
Beig, N. [28]	2020	Yes	T1, CET1, FLAIR	Manual	(i) tumor necrosis, (ii) enhancing region of the tumor, and (iii) peri-tumoral edema	Publicly available datasets; The Cancer Imaging Archive (TCIA) and Ivy Glioblastoma Atlas Project (Ivy GAP)	Image processing: https://github.com/MIC-DKFZ/HD-BET and https://github.com/MIC-DKFZ/nnUNet; R analysis code not publicly available
Verma, R. [29]	2020	Yes	CET1, FLAIR, T2	Manual	as above	Publicly available datasets; Cancer Imaging Archive, Cleveland Clinic, and Ivy Glioblastoma Atlas Project (GAP)	X-tile software (https://medicine.yale.edu/lab/rimm/research/software/)
Wu, J. [27]	2020	Yes	PET CT	Consensus Clustering	Implement MSI matrix to generate 3 habitats + spatio-temporal habitat evolution matrix to evaluate habitat evolution	Dataset from Stanford University Medical Center	Matlab code not publicly available
da Silva Neto, O [30]	2019	No	DCE	Molecular Texture Descriptor (MTD) and Pathophysiological Texture Mapping (MPT)	Generating 9 habitats according to change of relative enhanced over time	Publicly available datasets; Quantitative Imaging Network Collections (QIN)	N/A
Stringfield, O [25]	2019	Yes	T1, CET1, FLAIR	Otsu	2-steps approach to set 2 Otsu thresholds + sub-divide clusters based on FLAIR being > or ≤ white matter	Publicly available datasets; Cancer Genome Atlas (TCGA)	Matlab code not publicly available
Xing, S et al. [31]	2018	No	T2, ADC, simDWI	Probabilistic Classification	Generating 5 habitats based on similarity to reference tissue	Collected from Montreal General Hospital; ask for availability	Matlab code not publicly available
Lee, J et al. [24]	2015	No	CET1, FLAIR	Gaussian Mixture	2-steps approach to generate 4 habitats	Publicly available datasets: Cancer Imaging Archive (TCIA) and Cancer Genome Atlas (TCGA)	Matlab code not publicly available
Zhou, M et al. [20]	2014	Yes	CET1, FLAIR, T2	Gaussian Mixture	2-steps approach to generate 4 habitats	Publicly available dataset; Cancer Genome Atlas (TCGA)	Matlab code not publicly available

## Habitat imaging and radiomics

Radiomics is another popular quantitative imaging technique, which seeks to analyze a plethora of quantitative features extracted from medical images. Unlike traditional size and intensity analysis, radiomics has the ability to detect high-risk regions that may otherwise go unnoticed. Consequently, radiomics finds extensive application in treatment planning, image guidance, and predicting treatment outcomes in radiation therapy. In an effort to maximize the benefits of both habitat imaging and radiomics, researchers are currently exploring methods of combining the habitat imaging and radiomics.

### Extracting radiomics features from tumor sub-regions

There are multiple scenarios in which radiomics and habitat imaging can collaborate. One of them is extracting radiomics characteristics from habitats rather than the entire tumor volume. In a study conducted by Wang X. et al. [33], the authors compared radiomics features derived from the entire tumor with those derived from habitat imaging. By employing the Otsu threshold to maximize the variance between groups, they divided the tumor region into a high-intensity sub-region and a low-intensity sub-region. The analysis demonstrated that features derived from habitat imaging proven to be superior predictive biomarkers compared to those derived from the entire tumor. Cho et al. [34] utilized the same approach of MR-based habitat imaging to delineate the heterogeneity of perfusion in breast cancer. Additionally, they constructed a habitat risk score (HRS) based on habitat-derived radiomics features to classify patients into high- and low-risk categories. Compared with other risk models, the combined habitat risk model showed the best performance in the validation cohort, especially showing better performance in predicting DFS in breast cancer patients. These studies emphasize the potential advantages of habitat imaging in enhancing the performance of radiomics features. Furthermore, other investigations have employed this technique to determine the presence of EGFR mutation [35], predict progression-free survival [36], identify high-risk habitats [37], and predict survival rates [38]. Aminu et al. [39] discovered that habitat imaging has shown high accuracy in diagnosing and predicting the severity of COVID-19 in cancer patients, and models tailored for cancer patients perform better than models for the general population. Habitat-based radiomics has the potential to help identify high-risk cancer patients who require urgent and intensive medical care, and to distinguish COVID-19 from other types of pneumonia. Chen L et al. [40]. proposed a habitat-based radiomics method for preoperative differentiation between non-small cell lung cancer (NSCLC) and benign inflammatory diseases (BIDs). Their findings revealed that a combination of habitat and non-habitat features yielded the most accurate results. Moreover, Ismail, M et al. [41] delineated pseudo-progression and tumor recurrence using radiomics features derived from 14 global and 16 local habitats.

In summary, the Habitat-based radiomics has proven to hold great potentials. This tailored approach significantly improves the predictive precision in oncology, as compared to traditional global radiomics.

### Generating habitats with voxel-wise radiomics features

In addition to extracting features from habitats, there are other ways to combine both techniques. One approach involves the application of voxel-wise radiomics features, which entails calculating outputs based on a small region surrounding each voxel. A comprehensive study conducted by Bernatowicz et al. [42] evaluated the repeatability and reproducibility of voxel-wise radiomics features in lung cancer patients and explored the impact of these features on the computation of imaging habitats and found that nine features showed high repeatability and reproducibility in test-retest images. Researchers then applied Principal Component analysis (PCA) to select the 5 most informative principal components (PCs) for generating imaging habitats. Imaging habitats generated using these robust components (5 PCs) has improved stability over habitats generated using all 9 features, maintaining the stability of their numerical and classification results in the face of image perturbations. From 5 PCs, nine voxel-wise radiomics features were determined to be repeatable and reproducible. Using the robustness of voxel-wise features to improve computation in the imaging habitat may help identify biomarkers that are more relevant to tumor biology and clinical outcomes, which is crucial for personalized medicine and precision treatment strategies. Researchers from University of Cambridge [43] proposed a fusion technology of radiomics-based habitat imaging and ultrasound (US). Their technique uses CT-based radiomics signatures and habitat imaging to guide US-guided targeted biopsies in patients with high-grade serous ovarian cancer (HGSOC). This advancement in technology allows for the development of a precise tissue sampling technique based on radiomics habitats.

In conclusion, several experiments have combined radiomics, both region-wise and voxel-wise, with habitat imaging. Their results demonstrate great diagnostic, evaluative, and instructive potential of this combination. Table 3 summarizes all studies in Sect. 5.

**Table 3:** Study list of applications mentioned in Sect. 5 that combines habitat imaging and radiomics. From DCE: wash-in map (Ein); Washout Map (Eout); washout ratio map (RWO). Simple Linear Iterative Clustering algorithm (SLIC).

Author(s)	Year	Cancer Type	Imaging Stage	Biomarkers	Clustering Method	Habitat Generation Method	Radiomics Features Application	Data Availability	Analysis Tool Availability
Aminu, M et al. [39]	2022	Lung (*N* = 527)	COVID-19 diagnosis	CT	SLIC	1-step approach to generate 6 habitats for individual and whole population	Extracting features from habitats	Publicly available dataset: Cancer Imaging Archive (TCIA) and RICORD dataset; MD Anderson available with request with reason	N/A
Cho, H.H. et al. [34]	2022	Breast (*N* = 455)	Pre-treatment	Ein, Eout, Rwo	K-means	1-step approach with k value from 2 to 32 over the entire cohort	Extracting features from habitats	Dataset from Samsung Medical Center (SMC) and Gil Hospital (GH) not publicly available; request with reasons	Matlab code not publicly available
Cao, R. et al. [35]	2022	Brain Metastasis (*N* = 188)	Pre-treatment	CET1, T2	Manual	Manually segmented whole tumor, intra-tumoral necrotic, and peri-tumoral edema area	Extracting features from habitats	Primarily from LA	Pyradiomics and R code not publicly available
Verma, R. et al. [36]	2022	Glioblastoma (*N* = 150)	Pre-treatment	CET1, FLAIR, T2	Manual	Tumor sub-compartment: enhancing tumor, peri-tumoral FLAIR hyperintensities, and necrosis	Extracting features from habitats	5 publicly available data sets from supplement-1	N/A
Wang, X.H. et al. [33]	2022	Serous Ovarian Cancer (*N* = 161)	Pre-treatment	PET CT	Otsu	2-steps approach to generate 3 habitats	Extracting features from habitats	Dataset not publicly available; request with reasons	Imaging processing with LIFEx software and ITK-SNAP; python code not publicly available
Yang, Y. et al. [37]	2021	Glioblastoma (*N* = 122)	N/A	CET1, FLAIR, T2	K-means	1-step approach with k set from 2 to 9 to find optimal number	Extracting features from habitats	Dataset publicly available; Cancer Genome Atlas GBM	automated algorithm (https://github.com/MIC-DKFZ/HD-BET) and nnUNet model (https://github.com/MIC-DKFZ/nnUNet); R code not publicly available
Chen, L. et al. [40]	2021	Lung (*N* = 317)	Pre-treatment	PET CT	K-means	1-step approach with k from 2 to 10 to determine the optimal number	Extracting features from habitats	Original distribution in supplementary material	PyRadiomics
Bernatowicz, K. et al. [42]	2021	Lung (*N* = 500)	N/A	CT	K-means	Principal Component analysis (PCA) applied to select 5 PCs from voxel-wise radiomics features.	Generating habitats using radiomics features	Dataset publicly available; National Biomedical Imaging Archive (TCIA); request with reasons: VHIO CT dataset	PyRadiomics and OpenREGGUI
Beer, L. et al. [43]	2021	Ovarian (*N* = 8)	Pre-treatment	CT, Ultra-sound	Gaussian Mixture	1-step approach with 6 PCs to generate 3 habitats	Generating habitats using radiomics features	Recruited from Cambridge University Hospital	Microsoft Radiomics App; Matlab code not publicly available
Ismail, M et al. [41]	2018	Glioblastoma (*N* = 105)	Pre-treatment and Post-treatment	T1, T2, FLAIR	Manual	Expert delineation of the lesion habitat	Extracting features from habitats	Dataset from Cleveland Clinic and Dana-Farber/Brigham and Women’s Cancer Center	Matlab code not publicly available
Zhou, M et al. [38]	2017	Glioblastoma (*N* = 52)	Pre-treatment	CET1, FLAIR, T2	Otsu	2-steps approach to generate 4 habitats	Extracting features from habitat	Dataset publicly available Cancer Genome Atlas (TCGA)	Image registration by MIPAV medical image analysis software; code not publicly available

## Technical challenges

Summarizing the studies in previous sections, there are some considerations about challenges relevant to habitat imaging studies.

### Image registration and resampling

As a multi-parametric imaging technique, highlighted in previous sections, habitat imaging needs multiple biomarkers from multiple images as the input. Therefore, precise image registration to align the various images, within a single MR imaging session or cross modalities, is crucial for generating valid imaging habitats. Unfortunately, this registration process can distort voxel values, making it less than ideal. Nonetheless, it has been universally employed in many studies, particularly those utilizing a one-step solution. Seeking a way to avoid or at least minimize this distortion would help in generating more accurate habitats. Thus, it is necessary to choose images that are less affected by deformable registration. Images that are particularly prone to distortions, such as those acquired near air-tissue boundaries or in regions with complex anatomy, are more challenging for registration algorithms. For example, susceptibility artifacts near the sinuses or brain can cause local distortions that may be misinterpreted by the registration process. Similarly, motion artifacts due to unsteady breathing or cardiac motion can introduce inconsistencies in the image content, leading to misalignments.

Low-resolution images also pose a challenge for deformable registration because the lack of the fine details necessary for accurate feature matching and alignment. Additionally, images with low contrast between different tissues can also make it difficult for the registration algorithm to identify corresponding points or edges, potentially leading to registration errors. High noise levels in the images can further disrupt the feature detection process, which is fundamental to successful registration.

On the other hand, images with high resolution, high contrast, and low susceptibility to artifacts are generally less affected by registration errors. High-resolution images provide more detailed features that facilitate more accurate registration. Clear distinctions between different tissues, as seen in T1-weighted spin-echo images, make it easier for the algorithm to identify corresponding points and edges. Furthermore, images with reduced motion and noise, achieved through techniques such as cardiac or respiratory gating, produce cleaner images that usually lead to more reliable registration.

To mitigate the impact of these challenges, several strategies can be employed. For example, the use of fat suppression techniques or advanced coil technology can help reduce susceptibility and motion artifacts. Image enhancement through filtering or denoising algorithms can also improve the quality of the images before registration, reducing the impact of noise. Advanced registration algorithms that are robust to artifacts and can better distinguish true deformations from image distortions can be utilized. Employing multimodal registration, which incorporates additional imaging modalities or sequences, can provide complementary information to improve the registration process. The recent developed feature-based registration, using distinct features within the images that are less affected by artifacts, can also enhance registration accuracy. Finally, applying regularization techniques to constrain the registration to biologically plausible deformations can reduce the influence of artifacts on the registration outcome. Another possible solution is the two-step method, in which biomarkers are clustered prior to generating the final habitat map by identifying to which intersection of all individual biomarker habitats each voxel belongs. Registration is then applied to the clustered data to minimize the influence of abnormal values caused by distortion or artifacts.

### Choosing biomarkers

One limitation of current studies on MRI-based habitat analysis is that they often use structural images, such as T1- and T2- weighted images, which were primarily designed to show anatomical structure. However, the intensity of these MRIs can be influenced by various external factors, such as magnetic field inhomogeneity, receiver coil properties, scaling factors, and image acquisition parameters. These factors cannot be easily corrected or removed through intensity normalization [44]. For instance, researchers at Tangdu Hospital [37] used contrast-enhanced T1-weighted imaging (CET1) and T2-weighted fluid-attenuated inversion recovery imaging (FLAIR) to delineate edematous regions. Following K-means clustering, radiomics features were extracted for each clustered habitat, and radiomics signatures (RadScores) were constructed using these features. However, MRI sequences used in the Tangdu study could be inconsistent for each patient, potentially impacting the model’s validation performance. Moreover, this inconsistency hinders the implementation of group-level clustering as elaborated in Sect. 6.3. A more reliable, potentially more meaningful approach is to utilize imaging biomarkers that carries physiological information, often derived from advanced quantitative MR techniques, such as DWI, DCE, etc. Parametric maps derived from these MR sequences provide a better representation of the habitat. In clinical contexts, with a goal of developing tools for the assessment of treatment efficacy and the detection of resistant regions, the employment of physiological biomarkers is strongly encouraged. Furthermore, it is important to consider external validation and to make imaging techniques available at different centers. These are essential for further strengthening different successful habitat imaging studies and their conclusions.

### Clustering by group or individual

Clustering based on group level or individual level is an important choice to be made before any habitat imaging analysis. In certain studies [21, 22], the generation of habitats was predicated on individual-specific characteristics, reflecting a personalized approach to understanding tumor heterogeneity, The biomarkers of each patient were analyzed using the K-means clustering algorithm to identify habitats within his/her tumor. The rationale behind this choice is that structural MRI scans, such as T1- and T2-weighted images, exhibit significant variability across different patients and scanners, thus making it challenging to compare between different patients. However, given the inter-patient heterogeneity, the clustering and thresholding approaches would differ for different individuals. To derive a model with broader generalizability, aggregating data through cohort-wide clustering can illuminate underlying commonalities across the patient population. Choosing group-level clustering not only streamlines retrospective analysis but also enhances the capacity for prospective investigations. Therefore, for the sake of consistency and reliability, prioritizing group-level clustering with proper image pre-processing to minimize imaging inconsistency is strongly encouraged.

## Pathological validation and treatment evaluation

Researchers still need to understand the correlation between imaging habitats and pathological or even genetic information. Several studies performed their investigation from different aspects.

Du et al. [45] discovered that clustering parameters such as Calinski-Harabasz Index can be used to predict gene mutation. Their results showed that the difference between clusters and the similarity within clusters can reflect the mutation of the BReast CAncer gene 1 (BRCA1) in breast cancer. In this study, researchers also explored multiple parameters based on imaging habitats such as Calinski-Harabasz Index and Silhouette coefficient to evaluate the clustering result, and the ability of these parameters in predicting mutations through independent modeling or multi-parameter joint modeling. The results showed that the proposed model had good predictive ability and can be used as a powerful tool for clinical decision support. Syed’s [46] and Jardim-Perassi’s [47] group also demonstrated a significant consistency between imaging habitats and histological habitats. Furthermore, studies have shown a correlation between imaging habitats and tumor genetic information. For instance, Dextraze’s group [48] from the University of Texas investigated the correlation between tumor signaling pathways and imaging habitats. A total of 16 habitats were identified and certain habitats were associated with overall survival in GBM patients. Each habitat was found to be associated with unique pathway changes through Dirichlet regression analysis. This study reveals the clinical relevance of MRI-derived spatial habitats in GBM patients and their relationship to the molecular mechanisms of tumor biology.

In conclusion, many researchers have provided evidence that habitat imaging also measured genomic and molecular characteristics of tumors. Habitat imaging is able to delineate intra-tumoral heterogeneity from the fundamental aspect of tumor micro-environment. Table 4 summarizes the studies discussed in this section.

**Table 4:** List of pathological validation studies in Sect. 7

Author(s)	Year	Patient Details	Biomarkers	Clustering Method	Habitat Generation Method	Validation Method	Data Availability	Analysis Tool Availability
Du, T et al. [45]	2022	Breast (*N* = 187)	DWI, T1, Out-Phase T2, In-Phase T2, WA-TER T2, FAT T2	K-means	1-step approach to generate habitats	BRCA1 mutation status	Dataset from Second Affiliated Hospital of Dalian Medical University; request with reasons	Python code not publicly available
Syed, A et al. [46]	2020	Breast (*N* = 155)	ADC, kep, ve, Ktrans	Agglomerative	1-step approach to generate habitats	Histological assessment	N/A	Matlab code not publicly available
Jardim-Perassi, B V et al. [47]	2019	Breast (Mice)	T2map, T2*map, ADC, DCE-AUC, DCE-slope, DCE-time to maximum	Gaussian Mixture	2-steps approach to generate habitats	Histological assessment	Dataset from scanned mice	VisioPharm software for physiological analysis; ParaVision and Matlab code not publicly available for imaging analysis
Dextraze, K et al. [48]	2017	Glioblastoma (*N* = 85)	T1, FLAIR, post-contrast T1, T2	K-means	1-step approach to generate habitats	Tumor signaling pathways	Dataset publicly available: Cancer Genome Atlas	BraTumIA for segmentation; R code not publicly available for analysis

### Clinical applications in treatment evaluation

The utilization of imaging habitats in the field of radiation therapy presents a promising avenue for various applications. One such application involves the use of imaging habitats to predict treatment outcomes following radiotherapy, including factors such as tumor recurrence and survival rates. The response exhibited by tumors post radiotherapy can greatly differ among patients as well as within different regions of a single tumor. This variability poses challenges in ensuring the efficacy of radiotherapy. However, by employing non-invasive techniques, such as habitat imaging, to quantify and map tumor heterogeneity, it is possible to guide personalized radiotherapy and potentially enhance treatment efficacy. In recent years, there has been growing attention towards investigating the predictive potential of habitat imaging. Typically, these studies attempt to correlate the imaging habitat or its changes with the outcomes of radiotherapy, such as survival or recurrence rates. In this section, we review the applications of habitat imaging in treatment evaluation.

One primary focus of treatment evaluation studies is to predict survival rates following radiotherapy. Park et al. [21] conducted a study to assess the correlation between changes in these habitats over time and progression-free survival (PFS) in patients with glioblastoma treated with concurrent chemoradiotherapy (CCRT). CBV and ADC were used to generate three habitats using K-means clustering: hypervascular cells, hypovascular cells, and nonviable tissue. They found that a short-term increase in both hypervascularity and hypovascularity cell habitats volume size was associated with a significant shortening of PFS after CCRT. By combining these findings with other clinical predictors, they developed a habitat risk score that could stratify patients into categories of short, intermediate, and long PFS. Overall, they found that an increasing hypovascular habitat was the most predictive indicator of tumor progression sites. Beig et al. [28] also developed a survival risk score using radiomics features and habitats. They utilized gene set enrichment analysis to identify molecular signal pathways, providing a biological basis for the radiomics features. Their study successfully built predictive risk scores from habitats and demonstrated their association with signaling pathways related to treatment resistance. Many researchers have also confirmed the predictive ability of habitats for PFS using various techniques such as radiomics features [29], Local Binary Patterns [28], and habitat volumes [25, 27]. Moreover, Lee et al. [24]. utilized imaging habitats not only to predict 12-month overall survival but also to identify tumors driven by the epidermal growth factor receptor (EGFR).

In addition to survival rate, habitat imaging has been utilized in various aspects of treatment evaluation. Slavkova et al. [18]. developed mathematical models to simulate the growth of imaging habitats with and without radiotherapy. They identified three habitats with distinct image characteristics and developed a model family consisting of three coupled ordinary differential equations (ODEs) based on these habitats. Models were fitted to series of habitat maps at different time points of the control group and the treated rats to assess its predictive ability. They concluded that it is feasible to mathematically describe habitat dynamics in preclinical models of glioma using biologically based ODEs. Other researchers have also found that habitat imaging can predict tumor recurrence. Lee et al. [15] discovered that an increased hypovascular habitat volume fraction was associated with an elevated risk of brain metastases recurrence and the site of recurrence is also strongly correlated with the habitat location. Additionally, Weinfurtner et al. [23] found that the percent tumor volume remaining (%VR) and percent habitat makeup (%HM) were correlated with percent tumor bed cellularity (%TC) as a pathological response to neoadjuvant therapy.

Moving beyond common cancer treatment application, Aminu et al. [38] applied habitat analysis to a cohort of leukemia and melanoma patients who suffered more severe complications due to COVID-19. They developed a technique to quantify complex infection patterns by dividing lung regions into habitats with distinct phenotypes. By analyzing the association between habitat characteristics and radiologists’ semantic reading, they found that certain habitat characteristics were significantly correlated with radiologists’ reading results. The habitat imaging method was found to be significantly superior to existing methods in diagnosis and prognosis estimation especially for cancer patients during pandemic.

In conclusion, studies have supported that habitat imaging can assist in treatment evaluation by predicting survival rate, recurrence, and pathological response. Table 5 provides a summary of the studies discussed in Sect. 7.1.

**Table 5:** List of treatment evaluation studies in Sect. 7.1. (%) = percentage; PSF = progression-free survival; OS = overall survival; EGFR = epidermal growth factor receptor; %VR = percent tumor volume remaining; %HM = percent habitat makeup; %TC = percent tumor bed cellularity

Author(s)	Year	Patient Type	Stage of Imaging	Measurements from Habitats	Clinical Endpoint
Slavkova et al. [18]	2023	Glioma (*N* = 21)	Pre-, mid-, post-treatment	Model prediction of habitat volume	Habitat volume
Aminu, M et al. [39]	2022	Lung cancer (*N* = 527)	COVID-19 diagnosis	Radiomics features from habitats to train deep learning models	Covid-19 infection and severity
Lee et al. [15]	2022	Brain metastases (*N* = 83)	Post-treatment	%Habitat volume	Recurrence
Weinfurtner et al. [23]	2022	Breast tumor	Pre- and post-treatment	%TC, %VR, and %HM	Pathological response
Park et al. [22]	2021	Glioblastoma (*N* = 97)	Post-treatment	%Habitat volume	PFS
Beig et al. [28]	2020	Glioblastoma (*N* = 203)	Pre-treatment	Radiomics features from habitats	PFS
Verma et al. [29]	2020	Glioblastoma	Pre-treatment	Radiomics features from habitats	PFS
Wu et al. [27]	2020	Oropharyngeal squamous cell carcinoma (*N* = 162)	Pre-, mid-, post-treatment	Habitat volume	PFS
Stringfield et al. [25]	2019	Glioblastoma (*N* = 44)	Pre-treatment	%Habitat volume	PFS
Zhou et al. [38]	2017	Glioblastoma (*N* = 52)	Pre-treatment	Local binary patterns from habitats	PFS
Lee et al. [234]	2015	Glioblastoma (*N* = 65)	Pre-treatment	Spatial diversity features	OS and EGFR-driven tumor

### Longitudinal analysis

In the context of longitudinal studies, tumors may undergo significant macroscopic morphological changes due to treatment or progression, which poses challenges in image registration between scans obtained at different time points, necessitating the identification of consistent habitats across various time points. Despite these challenges, habitat imaging bypasses the need for temporal image registration, instead focusing on the consistency of the habitat generation process. The foundation of habitat imaging in these research lies in its ability to capture intrinsic tumor heterogeneity without the necessity of aligning images from different time points, emphasizing the importance of consistent habitat imaging biomarkers.

To maintain consistency of habitat generation is to keep biomarker units and clustering threshold identical. Weinfurtner et al. [23]. analyzed changes of percentage of habitat volumes between pre- and post-treatment images. Tumor segmentation was performed separately on images at two time points after clustering was performed based on the thresholds of the intensity distribution of the entire breast. In this case, temporal registration of tumor is not a necessary step for comparing percentage of habitat volumes. Certain studies have implemented group-level clustering to aggregate voxels from images across various time points. For instance, Wu et al. [27] conducted a group-level clustering on pooled voxels from both pre- and post-treatment PET and CT images. Despite the rigid registration performed on the pre- and post-treatment images, it was merely used to detect soft-tissue changes. Whether or not to do the registration does not compromise the outcomes of the habitat imaging analysis. This methodology, however, may not be directly applicable to MRI due to the susceptibility of MRI to various influences that can introduce variability in images obtained at different time points or on disparate devices. One potential solution is to employ identical MRI sequences at all-time points. Alternatively, quantitative parametric maps with definitive physiological meaning is recommended, as these are less likely to be affected by variations in scanning equipment or environmental conditions. Following a similar approach, Slavkova et al. [18] executed a group-level analysis prior to the application of ordinary differential equations without performing temporal image registration. The parametric maps being utilized to generate habitats include pharmacokinetic parameters K_trans_, v_e_, k_ep_, and ADC from DWI images.

In summary, temporal image registration is not a necessary step for longitudinal analysis. Typically, it suffices to apply uniform standard biomarker units for habitat generation. Researchers are encouraged to employ identical scanning protocols at each time point. MRI-based parametric maps are often favored due to the enhanced assurance of their consistency. By integrating these methodological strategies, researchers can potentially gain a better understanding of tumor dynamics over time and refine the evaluation of treatment efficacy.

### Ongoing open clinical trails

With the spatialized intra-tumoral heterogeneity provided by habitat imaging, some clinical trials are working on advanced radiation therapy planning strategies in order to take advantage of these information. Here, we list some of these studies with ID numbers from public records:

- NCT05301283: This trial investigates the use of MR-guided radiation therapy (MRgRT) for locally advanced pancreatic cancer. MRgRT can provide high precision in targeting the tumor while considering intra-tumoral heterogeneity. This can potentially improve treatment outcomes by adapting the radiation dose based on tumor response heterogeneity during the therapy course;
- NCT05868928: This trial focuses on improving the treatment of patients with non-small cell lung cancer using advanced imaging techniques to monitor tumor heterogeneity. The goal is to tailor radiation therapy more effectively by assessing changes in tumor characteristics over time, thus optimizing therapy based on individual tumor characteristics;
- NCT06002711: This trial aims to explore the use of imaging biomarkers to better understand tumor heterogeneity in prostate cancer. By utilizing multi-parametric MRI, the study seeks to enhance radiation therapy planning and delivery by identifying regions within the tumor that may require different radiation doses based on their heterogeneity.

## Discussion

Habitat imaging has been widely used in many aspects of cancer treatment. As a non-invasive tool to measure intra-tumoral heterogeneity, it has broad application prospects in personalized treatment.

One of the advantages of habitat imaging is its suitability and diversity in measurement. As a multi-parametric imaging technique, habitat imaging measures TME from different structural or physiological angles using corresponding imaging biomarkers. This characteristic of habitat imaging will expand its frontier with advancements in medical imaging techniques. Other quantitative imaging techniques, such as radiomics features, can also be used as input biomarkers for habitat imaging. Therefore, it is recommended to explore and incorporate more techniques as input biomarkers to maximize the potential of habitat imaging. Researchers should also focus on methods of habitat generation. While the automatic clustering method is popular in many studies, further efforts are needed to compare the accuracy and efficiency of the two approaches mentioned in Sect. 4. Additionally, researchers should investigate and optimize habitat generation methods, as well as explore their combination with imaging techniques such as radiomics.

Habitat imaging also faces several technical challenges that require attention. The alignment of multiple image modalities before habitat analysis often necessitates image registration and resampling. However, this process can introduce distortions in voxel values and affect measurement accuracy. It is crucial to find a way to either bypass this process entirely or minimize its impact. Another important consideration is the selection of biomarkers, as the intensity of structural MRI sequences is typically influenced by external factors that are difficult to correct. On the other hand, MRI sequences that can provide physiologically meaningful biomarkers are recommended to be included for habitat imaging. These physiological biomarkers are particularly beneficial for multi-center and group-level clustering studies, for both retrospective and prospective data analyses.

Recent studies have also revealed correlations between imaging habitats and pathological or even genetic information, thereby validating the ability of habitat imaging to measure tumor heterogeneity from a fundamental aspect of the tumor micro-environment. Researchers have explored the clinical applications of habitat imaging for treatment evaluation, and their results demonstrate its predictive ability in various aspects such as survival rate, recurrence, and pathological response. Although glioblastoma has been the most extensively studied cancer so far, further studies are needed on the application of habitat imaging to other types of cancers.

### Preclinical and clinical studies

Preclinical and clinical studies in this review were summarized in Table 6. Overall, the number of preclinical studies is lower as compare to clinical studies. Preclinical research primarily encompasses longitudinal studies or pathological analyses, which are characterized by higher costs and stringent requirements for standardization when implemented on human subjects. These studies may involve extended observation periods or detailed examination of disease progression, which can be resource-intensive and require meticulous methodology. On the other hand, clinical research is predominantly retrospective, often focusing on the analysis of imaging data to draw conclusions about patient outcomes and treatment efficacy. This approach is often more feasible in terms of cost and accessibility. The focus on imaging analysis in clinical research allows for the evaluation of treatment responses and the identification of prognostic indicators without the need for additional invasive procedures or high costs associated with experimental interventions. In the future, we will likely see an increased emphasis on prospective clinical trials to validate the effectiveness of habitat imaging methods.

**Table 6:** List of preclinical and clinical studies

Category	References
Preclinical Studies	Slavkova, K. P. et al. [18], Syed, (A) K. et al. [46], Jardim-Perassi, (B) V. et al. [47]
Clinical Studies	Kazerouni, A.S. et al. [19], Zhou, M. et al. [20], Park, J. E. et al. [21], Park, J. E. et al. [22], Weinfurtner, R. et al. [23], Lee, J. et al. [24], Stringfield, O. et al. [25], Wu, J. et al. [27], Beig, N. et al. [28], Verma, R. et al. [29], da Silva Neto, O. P. et al. [30], Xing, S. et al. [31], Tar, P. D. et al. [32], Wang, X. et al. [33], Cho, H.-h. et al. [34], Cao, R. et al. [35], Verma, R. et al. [36], Yang, Y. et al. [37], Zhou, M. et al. [38], Aminu, M. et al. [39], Chen, L. et al. [41], Ismail, M. et al. [41], Bernatowicz, K. et al. [42], Beer, L. et al. [43], Nerland, S. et al. [44], Du, T. & Zhao, H. [45], Dextraze, K. et al. [48]

## Conclusion

Habitat imaging, as an emerging technique, holds great potential as an effective method for assessing tumor biology and intra-tumoral heterogeneity in an explainable way. However, the current literature on habitat imaging applications is limited by the types of biomarkers, clustering methods, and lack of pathological validation. It is essential to conduct further research to validate this technique and explore a broader range of applications. To achieve this goal, additional studies should focus on investigating clustering techniques, exploring new biomarkers, assessing reproducibility, combining it with other techniques like radiomics, and histological validation. Overall, habitat imaging has great potential in guiding personalized cancer treatment especially radiotherapy to overcome the challenges posed by tumor heterogeneity.

## Data Availability

No datasets were generated or analysed during the current study.
